# A child in pain: A psychologist’s perspective on changing priorities in scientific understanding and clinical care

**DOI:** 10.1002/pne2.12034

**Published:** 2020-08-04

**Authors:** Kenneth D. Craig

**Affiliations:** ^1^ University of British Columbia Vancouver BC Canada

**Keywords:** assessment, biopsychosocial, communication, facial expression, socialization

## Abstract

My research and clinical career followed a trajectory of increasing appreciation for the importance of social factors as determinants of pain experience and expression. The social contexts of children’s lives determine whether infants and children are exposed to pain, how socialization in family and ethnocultural contexts lead to pain as a social experience, comprised of thoughts and feelings as well as sensory input, how others shape pain experience and expression, less so for automatic/reflexive features than purposeful representations, and how other's appraisals of children’s pain reflect the observer's unique background and capacities for intervening in the child’s interests. A greater understanding of the social dimensions of pain, as reflected in the social communication model of pain, would support innovation of psychological and social interventions.

## 1 INTRODUCTION

A child is in pain! What is the matter? What to do? The likely and expected response would be to react with alarm, to concentrate on discovering why the child is in distress, to protect the child from harm, and to provide comfort, but I am uncertain these are always, or even often, accomplished. This arises from personal family experiences, work in clinical settings, and long exposure to research on pain in childhood. There is too much evidence of unrecognized pain, willingness to ignore children’s pain, inadequate assessment, underestimation of the child’s distress, and inadequate or faulty management.

What should our priorities be in undertaking research and care for pain in children? Unquestionably, we need to know more about the biology of pain and the injuries and diseases that precipitate states of painful distress in children, including the rapid neurobiological and developmental changes in how children experience pain as they mature through childhood, and we need better biologically and psychologically oriented interventions able to provide relief. But we also need to know more about the interpersonal phenomena implicit in the issues raised in the first paragraph above. Our understanding of pain is incomplete, and our ability to help children is impaired without a greater knowledge of social factors in pain.

Social parameters of pain are the poor cousin of an overwhelming current focus upon biological mechanisms and treatment of pain. Healthcare budgets, research granting agencies, educational curricula, textbooks, journals, and conference programs focus heavily upon physiological and neurochemical processes. Social features of pain remain relatively unexplored and often ignored even though they may be central to recovery and ongoing function.^1^

This paper responds to the invitation to reflect upon my research in the context of the development of the field, both the past and likely future developments. I explore my efforts over several decades to understand social determinants of pain and pain‐related disability in infants and children and their role in pain control and rehabilitation.^2^ My work has been motivated by concern for children’s suffering and its long‐term consequences for the child, their family, and the community at large. As well, pain in its manifest complexity is a fascinating intellectual and scholarly puzzle that slowly is yielding to understanding through application of the scientific method, although novel, effective therapies have been slow to emerge. Fortunately, there have been accelerating rates of research on pain in children.^3^

## 2 AUTOBIOGRAPHICAL MUSINGS

Given that life history determines ongoing interests and pursuits, some background observations seem in order. I was a graduate student in clinical psychology at the University of British Columbia, Purdue University, and the Oregon Health and Sciences University at a time of transformation of this discipline from a theoretically driven profession to one that emphasizes evidence‐based care. My PhD research supervisor, Fred Kanfer, was a leader in the field.^4^ Interpersonal dimensions of mental health phenomena were my earliest research interests,^5^ but this rapidly became interests in empathy for the distress of others^6^ and the impact on pain of observational learning,^7^, ^8^ the process whereby a person’s behavior changes as a product of observing others responding to their social and physical environments.^9^, ^10^ I am also grateful that psychologists such as Ronald Melzack,^11^ Bill Fordyce,^12^ and Richard Sternbach^13^ made clear the importance of psychosocial studies of pain.

There were many opportunities to advance understanding through research on children’s pain in the 1970s,^14^ given both a paucity of research‐based evidence and many myths concerning children’s pain at the time. For example, there were claims in the psychological, medical, and nursing literatures to the effect that brain and neural systems were insufficiently developed to support pain experience in the newborn, and, even if they could experience pain, they lacked a capacity to remember the experience. There also was a fear of the side effects of analgesics.^15^ Fortunately, this dismissal of the possibility of infant pain now has been discredited by substantial biobehavioral research that demonstrated massive neurobiological and stressful impacts as well as the long‐term deleterious impacts of uncontrolled, repetitive pain exposure in preterm, term, and older infants on cognitive, emotional, and social functioning.^16^, ^17^, ^18^

A particular interest in socialization of pain experience and expression during early childhood^19^ and an appreciation for ethological methods, that is, detailed unobtrusive observation of the phenomena of interest in natural settings, for example, pain in the clinic or at home, led to detailed coding of infant’s reactions to immunization injections in preventive health clinics.^20^ Initially, we observed changes over the first 12 months of life from a spontaneous, diffuse reaction to evidence of anticipatory distress, beginning use of meaningful language, and goal‐directed movements. Unobtrusive observation and behavioral coding of infant and children’s pain events in life settings and the reactions of parents and others became a favored research method in understanding pain as a contextualized experience.

There also was an eureka moment that led to strong convictions concerning the value of the experimental method in studying social parameters of pain. Studies of pain empathy had indicated that observing others in pain typically evoked powerful physiological reactions that differed from the physiological reactions to painful events themselves,^21^ studies that presaged current work using brain imaging to contrast personal and vicarious experiences with pain.^22^ During a laboratory meeting, it was suggested that variations in how people reacted when in pain might have an enduring impact on observers. Indeed, we found a dramatic impact of variations in social models’ tolerance of pain on people’s self‐reports of pain and willingness to tolerate noxious stimulation—people exposed to others who were highly tolerant of pain came to present themselves in this manner, likewise, exposure to intolerant people led to a pattern signifying pain intolerance.^23^, ^24^

This was supported by replications and variations pursuing other dimensions of social influence on pain (eg, Craig).^8^, ^25^ The findings were impressive, but the use of self‐report as an index of pain did not lead to confidence that social influences changed the experience of pain—perhaps observers were driven by social motives or impression management to match the models, say, an unwillingness to disagree with social peers. Should one accept self‐reports as exclusively reflective of experienced pain? This led to a series of studies examining whether exposure to variably sensitive social models would have a comparable impact on measures of pain experience that were less vulnerable to socially motivated misrepresentation. In brief summary, we found social models to have an impact on psychophysiological measures, psychophysical measures of sensory sensitivity, sensory decision theory measures of sensory sensitivity and response bias, and nonverbal measures of pain (all summarized in^26^). These controlled studies provided considerable confidence that the immediate social environment had a substantial impact on fundamental features of the experience of pain. I was also struck by the power of the experimental method. Findings reveal universal truths, at least until somebody else discovers otherwise.

From here, the research pursued multiple directions, inspired by questions provoked by the studies themselves, the enthusiasms of graduate students and postdoctoral fellows who came to work in the laboratory, and collaborations with colleagues in other laboratories. They are too numerous to note here, but several former graduate students who have established highly productive laboratory groups and outstanding scholarly reputations require mention: Ken Prkachin, at the University of Northern British Columbia, Ruth Grunau, at the University of British Columbia, Heather Hadjistavropoulos, at the University of Regina, Rebecca Pillai Riddell, at York University, and Christine Chambers at Dalhousie University. Thomas Hadjistavropoulos also became a valued colleague as a research associate in the laboratory for several years. The following are some of the preoccupations I pursued with these scholars and others.

## 3 CONCEPTUAL MODELS

Conceptual models structure and guide thinking about the nature and management of children’s pain, yet they vary considerably in emphasis on psychosocial factors.^27^ Models are social constructs—invented for explanatory purposes, often fallible, and subject to change on the strength of better evidence.

The biomedical perspective has wide acceptance among healthcare professionals and the public alike, because it coheres with most people’s experiences of pain and it certainly has contributed to our understanding of the biology of pain and biomedical treatments. Virtually, everybody has learned about acute, phasic pain through the commonplace minor injuries of childhood. These experiences provide a basis for beliefs that pain is caused by tissue damage and will resolve with healing and medical care. Unfortunately, the biomedical model fails many people—it has long been recognized as insufficient^11^, ^28^—painful experience is only modestly correlated with tissue damage, acute pain does not always abate, tissue damage in the form of injury or disease often cannot be found, despite the best diagnostic efforts, and the best biomedical interventions, pharmaceutical, surgical, and otherwise, are only partially successful. Conceptual models of pain that incorporate psychological and social constructs now are recognized as having superior explanatory power.

Careful research demonstrates potent roles for psychological mechanisms in understanding the dramatic variations in painful response to injury or disease, and social factors are recognized as potent determinants of pain in children and adults.^1^, ^2^, ^29^ Pain varies with how people think and feel as well as with sensory input and pain is a contextualized experience—variable depending upon the social and physical setting in which it occurs.

Human pain is complex, in part because we evolved as social animals—we are hard‐wired to be connected to others and to be influenced by them, even in such fundamental processes as sensory experience. Whether the evolutionary changes leading to human capacities for complex cognitive processing incorporating the social context were a consequence of the challenges of life in competitive and cooperative societies or were a consequence of other evolutionary processes, human brains support social features of pain experience, along with primordial sensory and emotional features that appeared, and were conserved in ancestral species. Understanding the biology of pain in humans and progenitor species will require incorporation of a social neuroscience perspective on how the brain encompasses social dimensions in the perception and meaning of pain.

One focus in our efforts to broaden thinking about pain has been the inadequacies of the 1979 International Association for the Study of Pain (IASP) definition of pain. For example, Anand and Craig^30^ noted that the emphasis on self‐report in this definition and its notes tended to exclude consideration of nonverbal populations (eg, infants, young children, children with intellectual disabilities, people with cognitive impairments). IASP was responsive to the criticism and subsequently added a note to the definition, indicating, “The inability to communicate verbally does not negate the possibility that an individual is experiencing pain and is in need of appropriate pain‐relieving treatment.” Subsequently, Williams and Craig proposed^31^ updating the IASP definition to recognize decades of research leading to our evolved understanding of pain. Definitions have important consequences—they direct attention to key features of the phenomena. The narrow focus on sensory and emotional features in the IASP definition supports biomedical interventions well, but fails to acknowledge the importance of cognition and social experience as defining features of this complex experience. Making these salient adds explanatory power and supports widely adopted and newer multidisciplinary approaches which match treatment to children’s personal and social characteristics, an approach consistent with the biopsychosocial model of pain. The updated definition reads, “Pain is a distressing experience associated with actual or potential tissue damage with sensory, emotional, cognitive and social components.” This proposal has been contentious,^32^, ^33^ and there has been considerable debate with other proposals being considered.^34^

## 4 ASSESSMENT AND MEASUREMENT

There can be no scientific or clinical progress without measurement tools capable of documenting the phenomena of interest. Pain is challenging because it is inherently personal, largely private, and often concealed, as people attempt to maintain control and be socially appropriate. But all people have great interest in whether others are in pain and the sufferer may, but not always, benefit if others are aware, particularly when there is clinically significant, sudden onset acute pain or acute exacerbations of chronic pain.^35^

In reality, abundant data concerning the response of the person in pain tend to be available to observers, whether untrained family, friends, or strangers or healthcare professionals. Some of the complex observable features of pain reactions are quite automatic, reflexive, or unconscious, such as withdrawal reactions from threatened or real tissue injury, some involuntary vocalizations, or, most often, facial grimaces. Other features of the reaction are more purposeful, intentional, or conscious, as people attempt to engage others or protect themselves using language or organized behavior such as refusing to engage in work or other activities.^36^, ^37^ The involuntary/controlled distinction is consistent with our understanding of fundamental dual neuroregulatory systems in complex biological organisms.^38^ The sensory/discriminative and emotional features of the experience are largely regulated by an involuntary system, but pain is not purely a physical sensation—this information is decoded and processed in the context of memory and social cues to yield the subjective experience. Observers of others in pain intuitively make this distinction,^39^ and it is well‐represented in items on nonverbal observational scales designed to assess pain.^40^, ^41^

Of this wealth of information, use of one medium of communication, self‐report, predominates as the preferred strategy in both basic and applied pain research,^42^ often justified by the remark that it is “the gold standard.” While nuanced description of painful experiences can be very informative and it is important to validate children’s distress, self‐report as usually practiced has notable limitations and may not lead to improved pain management.^43^ It is misguided to believe in a one to one correspondence between the felt experience of pain and self‐reports. People, including children, are sensitive to social contexts and typically predicate voluntary reports upon their perceived best interests, with reports varying with the audience.^44^ Children are not only capable of misrepresentation, suppression, or exaggeration, but many will admit to having done so.^45^, ^46^ Self‐report also requires extensive cognitive, linguistic, and social competence. In the case of pain, these capabilities reflect life histories of socialized sensitivity to noxious somatosensory experiences. For example, others serve as role models and their actions may reinforce substantial or diminished pain reactivity,^47^, ^48^ with this affecting perceptual and psychophysiological properties of the experience.^49^

The self‐report scales used extensively in clinical and research settings also have real limitations.^42^ Pain is recognized as multidimensional, yet the majority of scales use global estimates of pain intensity^50^ that obscure the richness of the experience^51^ and sacrifice the potential for interventions varyingly targeting cognitive, emotional, or social features of the experience.^52^ As well, pain ratings can be biased by modest changes in instructions or subtle differences in scale items.^53^ For example, ratings on scales asking the child to select one of a range of facial displays that best represents their current experience are biased toward more severe ratings if the lowest item on the scale depicts a happy face.^54^ Children who are not in pain are not necessarily happy.

Nonverbal expression is the other conspicuous category of behavioral information available to observers, including facial expression, limb movements, posture, gait, or paralinguistic qualities of speech. Most salient is facial expression, a powerful and important communication medium, with observers able to discern not only pain, but emotional and motivational states, attention, interest, etc.^55^, ^56^, ^57^ It usually is available spontaneously and continuously, neither requiring voluntary expression nor prompting, it appears less subject to voluntary control, thereby more likely to represent “felt” rather than dissembled pain, and it does not require cognitive, linguistic, or social competence for expression, hence, it is available in populations without verbal communication competence.^58^ Facial displays also provide opportunities to differentiate pain from other states of distress,^59^ if detailed analyses are undertaken.

Our capacity to assess pain in infants and children was advanced substantially by Ruth Grunau’s decision to examine pain expression in neonates using facial expression and cry.^60^ We adapted characterizations of the relatively stereotypic facial expression of pain in adults^55^ to the unique physiognomy of the infant face. The resulting Neonatal Facial Coding Scale (NFCS) was: (a) reliable and valid for both term and preterm infants,^61^, ^62^ (b) responsive to variations in sleep/waking behavioral states in infants, indicating integration of the expression with fundamental biological systems,^60^ and (c) reflective of developmental changes during infancy.^63^, ^64^

Facial expression has come to be used extensively in clinical and basic research examining biological, behavioral, and social features of pain. The NFCS infant scale continues to be subject to study and refinement,^65^, ^66^ and it has provided a basis for study of contextual^67^ and modulating factors that determine pain expression and developing capacities for self‐regulation of pain and stress.^68^, ^69^ It also has proven to be a valid and useful outcome measure for investigating analgesic interventions.^70^, ^71^, ^72^ Many observational pain assessment instruments for preschool and school‐aged children depend upon facial expression.^73^ Regrettably, characterizations of features of the facial displays on these scales often differ from that which is empirically observed, leading to reduced reliability and sensitivity and greater risk of observer bias.^74^

It is noteworthy that this work on facial expression in the assessment of pain in infants and young children provided a solid foundation for investigations of pain in other populations incapable of self‐report,^58^ including children and adults with intellectual disabilities,^75^ children with autism,^76^ youths with significant neurological impairments,^77^ and persons with dementia.^78^ Studies using objective facial coding measures have frequently contradicted claims that certain populations were insensitive or indifferent to pain based upon subjective, global impressions, and anecdotal accounts.^79^

The study of infant facial expression also inspired interest in pain communication in nonhuman animals, with our development of the mouse grimace scale^80^ leading to similar scales for the study of pain in animals such as rats, rabbits, horses, cats^81^ and sheep, pigs, ferrets, and seals,^82^ developments of great interest to scholars in veterinary science and animal welfare. The facial grimaces observed tend to represent combinations of actions common across species and species‐specific actions, reflecting evolutionary conservation along with species‐specific adaptations. Given that this measure can examine clinically important spontaneous pain associated with injury or disease, rather than pain instigated by laboratory devices, it has the potential to change use of analgesics with animals.^83^

Given that pain is encoded in relatively stereotyped facial displays, there has been strong interest in the application of computer vision, pattern recognition, and machine learning strategies to the assessment of pain.^84^, ^85^ This artificial intelligence approach has the potential to circumvent biases in reporting pain and in judging pain in others. We have found the strategy effective in postoperative pain assessment in children and youth.^86^, ^87^

## 5 LEARNING THE EXPERIENCE OF PAIN

As noted above, widely endorsed definitions of pain ignore the important role of cognition. Yet, pain is a conscious experience, a perception rather than a sensation with emotional overtones, and personal understanding and control are enhanced by social learning. Neither the sensory nor the emotional components would exist without conscious awareness and personal appreciation for what is happening. Cognition governs the transition from nociceptive input to subjective pain. While the complex biological response to tissue insult is largely outside of awareness, adaptive functions are fulfilled through understanding, deliberation, and problem‐solving in organisms capable of cognitive processing, humans in particular. Organisms that do not have evolved cognitive capacities rely upon reflexive and automatic biological systems. More sophisticated brains permit organization and use of complex patterns of information. We have argued for understanding developmental trajectories, as recognition of primitive threats to physical integrity in newly born children^30^ become elaborated through physical maturation and life experience, including social learning, to create meaning and understanding.^88^

It is important to recognize that cognitive features of the experience are integral and immediate properties of the experience, rather than consequences of being in pain as was argued in the past. In 1968, Melzack and Casey presented a model of sensory, motivational, and cognitive components of pain, rejecting pain as a primary sensation temporally followed by motivational and cognitive reactions to pain sensation.^89^ Recent work using brain imaging supports this position by demonstrating that the “neurological pain signature” is associated with a pattern of concurrent activation of multiple regions in the brain and serial processes.^90^, ^91^ Pain immediately captures attention in conscious awareness, thereby engaging a complex apparatus of memories, organizational schemas, beliefs, and attitudes at the same time as there are somatosensory awareness and emotional arousal, all in the interests of escape, avoidance, or otherwise resolving current and further threat.

But what are the contents of these experiences? We learn from a life history of experiences with pain, our own, and those of others. Cognitive maturation intersects with social experiences to determine thoughts, expectations, appraisals, coping strategies, and emotional reactions. Intergenerational transfer of information and knowledge is of importance in all human learning, but no less so in the transfer of understanding and skills useful in controlling risks of physical danger.

Personal and vicarious experiences of pain are commonplace in young children,^92^ with evidence suggesting toddlers experience pain as often as hourly.^93^, ^94^ Exposure to the experiences of pain in others is far more common than personal experiences as children spend lots of time with their age peers. There is enormous opportunity to learn about what is potentially dangerous and variations in reactions of peers, parents and other adults instruct how different ways of expressing pain provoke different consequences. In this manner, children learn about normative family and ethnocultural patterns of response to pain, the consequences, favorable, and unfavorable, of responding in different ways, and the general impact of being expressive or stoical in pain expression.

The family is of major importance^95^ in the social learning of pain. Parents, siblings, and others provide considerable instruction through physical guidance, verbal coaching,^96^ and opportunities for observational learning.^8^, ^97^, ^98^ Conformity to patterns deemed socially appropriate and desirable would be reinforced. In this manner, a child’s unique personal history as well as patterns of thinking and behavior normative to the child’s family and cultural background will become reflected in how children respond. Similarly, families provide potent social models for emotional reactions during painful events. There now are many studies demonstrating that children’s risk for chronic pain is increased by the presence of a parent or other prominent family member who suffers persistent, recurrent or particularly painful bouts of pain.^99^, ^100^

Other theoretical and empirical approaches examine features of the complex dynamics of family influence on children’s pain. The application of fear‐avoidance models of pediatric pain chronification^101^ focuses upon children’s sensitivity to their parent’s perception of events as threatening or dangerous. Parents who experience high levels of fear and catastrophic thinking can engage in increased solicitousness that feeds into children’s appraisals in a progressively debilitating cycle. Children who are more inclined to engage in pain catastrophizing are more likely to report feelings of distress or higher pain intensity.^102^ The communal coping model proposes that children prone to high levels of catastrophizing about pain do so as a communicative appeal for support or empathy from others.^103^ The intricate complexities of caregiver self‐ versus other‐oriented goals and emotion regulation are featured in the Vervoort and Trost^104^ affective‐motivational model of interpersonal pain dynamics.

## 6 LEARNING TO EXPRESS PAIN

Engaging others in providing care when needed is a more complex task than is usually assumed to be the case. One expects sympathy and altruism would be reflexive and universal, but the data suggest people without expressive skills, particularly those in vulnerable populations, are less successful in attracting care than people who acquire a full complement of verbal and nonverbal skills.^58^

The capacity to effectively communicate painful distress includes skill in suppressing pain expression. Inhibition of pain expression may be important to personal safety, or, more prosaically, behaving in a socially desirable manner. There generally is a reluctance to admit to being in pain, perhaps perpetuated through the “don’t cry wolf” fable. Children are expected to learn to suppress expression of pain unless it is serious, although there are variations across ethnic groups.^105^ While protection from danger and delivery of care are important to families, communities, and institutions embedded within them, people learn that limited resources should not be exploited. This capacity to down‐regulate pain expression is complemented by a capacity to up‐regulate the expression.^106^ Concern about this can preoccupy people concerned about risks and costs of faked pain in the interests of compensation, litigations, and relief from occupational, domestic, and other life demands.^107^

There are risks attached to appearing vulnerable in the presence of enemies or strangers as well as expectations and constraints concerning appropriate display in social settings. The more automatic or reflexive pain expressions can be suppressed so as to be context appropriate, akin to how one can inhibit a cough, flatulence, or eliminative imperative, at least temporarily. This has been demonstrated for facial expression. While pain expressions are partially capable of being inhibited or faked, suppression is difficult and not wholly successful,^46^, ^108^ with subtle differences distinguishing fake displays from genuine expression^108^, ^109^—one distinction identified has been for the faked expression to be more exaggerated than the genuine expression. As well, efforts to suppress are only partially successful, with “leakage” of the underlying felt experience observed. These subtle differences represent “honest” evidence of painful distress for observers.

The more purposeful, intentional, conscious, or coordinated patterns of pain expression are more likely to be attuned to specific social contexts. In this sense, they would reflect exposure to idiosyncratic family, ethnic, and cultural norms. Progressive sophistication and social competence in the use of language have been documented.^110^, ^111^ Children’s vocalizations during pain transform from relatively reflexive expressions during infancy, crying, “ow” or “ouch,” to use of words like hurt or pain. As noted above, socialization in specific family, ethnic, and sociocultural contexts instructs both how one should think and feel pain and how to be socially appropriate in the expression of pain. Kunz et al^47^ effectively demonstrate how facial displays of pain are subject to operant reinforcement, consistent with social criteria for what should be displayed and when. Social display rules prevail in social settings. We learn when, where, and how to display affective states.^112^ In a seminal paper entitled “Display rules for anger sadness, and pain: it depends upon who is watching,” Zemen and Garber^113^ examined factors influencing children’s decisions to control or express their emotions, finding variability across the type of emotion, age, and sex. The expectation of a negative interpersonal interaction following disclosure was a primary reason for emotional control.^113^

## 7 OBSERVER JUDGMENTS AND DECISION PROCESSES

Observers motivated to understand and control pain in others are challenged by these complexities, among others. Observer reactions are consistent with dual neuroregulatory systems—they can be characterized as automatic and reflexive, the gut reaction to observing another’s painful distress,^114^, ^115^ or purposeful and reflective, as the individual contemplates what is happening and attaches meaning to the situation.^37^, ^116^ Individual differences in judgments also would reflect sensitivity, disposition, and skill, as well as personal experience and educational influences, making them highly personal.

The necessity of assessing pain using imperfect assessment instruments creates uncertainty^117^ and errors and systematic biases. These are a product of the interaction between use of observed events (bottom up information) and personal appraisals related to the observer’s beliefs, attitudes, prior history, and the relationship with the person in distress (top down processes).^118^ It is no small surprise that judgments often reflect factors unrelated to the person’s pain. A general tendency to underestimate or discount the pain of others^119^, ^120^ is tempered by similarities between the observer and person in pain, for example, gender, age, ethnicity, the extent of kinship or friendship,^121^ catastrophic thoughts or hypervigilance and fear for the other person,^122^ availability of a medical diagnosis to account for the pain, the presence of evidence that social or psychological factors are influencing the pain,^123^ and many other factors. The task seems formidable.

The potential for biased observer judgments and the risk of misrepresentation by the person in pain contribute to the interest of using computer vision, pattern recognition and machine learning algorithms to automate pain assessment using physiological and behavioral data.^86^, ^87^

## 8 THE BROADER PERSPECTIVE

One searches for an integrating conceptual model. Mixing biological, psychological, and social constructs together can be cumbersome, but ultimately necessary. The evolutionary perspective provides some basis for integration. Biological systems transform allowing adaptation to environmental demands. The origins of human pain are rooted in biological systems that emerged in ancient ancestral organisms, but these transformed to reflect different ecological niches during speciation, with human pain ultimately manifesting characteristics of human speciation, along with conserved ancestral characteristics.

Most efforts to provide overarching frameworks focus on one of these levels of analysis, including only general reference to other levels of analysis. Our approach has focused upon social factors. The Social Communication Model of Pain (Craig^124^, ^125^) provides a general framework designed to make social parameters of the biopsychosocial model of pain salient and meaningful.^1^ This paper has focused upon how pain is experienced and expressed and how others appraise and respond to the person in pain. No doubt this model will continue to transform. Models need to go beyond dyadic influences to consideration of the extended social ecological contexts of pain and care provision. Most human public and private institutions have a role in how well pain is understood and care is provided. The extent and quality of care is related to attitudes and structures in healthcare delivery systems, granting agencies, educational institutions for healthcare providers and others, and other agencies.

To illustrate, general compassion in a society is reflected in how well the community cares for vulnerable populations. This includes infants and young children, people with intellectual disabilities and cognitive impairment, and people who are marginalized in the community by social factors, including, but not always, people living in poverty, people with mental health problems, the homeless, those with substance abuse disorders or histories of incarceration, racial groups, people in the lesbian, gay, bisexual, transgender, transsexual, queer, and 2‐spirit (LGBTQ2S) community, refugees and recent immigrants, and others. These children and adults are subject to the same sources of pain in injury and disease as the population at large, but also as a result of stress and violence and other conditions unique to the population and lack of adequate care. Unfortunately, evidence has accumulated indicating restricted access to pain management in these populations,^33^ a pattern consistent with stereotyping, discrimination, stigma, and maltreatment. Focus groups representative of people in Indigenous, LGBTQ2S, and refugee and new immigrant populations suggest they are subjected to devaluing and discrediting treatment not only from people who could be characterized as antagonists or strangers, but also from friends, family members, employers, and healthcare professionals.

## 9 THE FUTURE

The research trends described above perhaps best signal my predictions for the future:

1. A greater willingness to embrace all aspects of the biopsychosocial model of pain, including serious exploration of the social determinants of pain in the interests of improved understanding and treatment development.
2. Greater use of a developmental perspective that recognizes the substantial and rapid transformations in pain associated with maturation and life experience.
3. A shift away from limited biomedical approaches to pain that focus on sensory features and efforts to control pain through biomedical interventions to consideration of the thoughts, feelings, and social lives of infants and children in understanding pain and treatment planning.
4. Use of a broader and more detailed model of pain in pain assessment that includes recognition of the importance of nonverbal pain expression and the multidimensional features of pain experience.
5. Use in treatment formulations of the natural socialization processes that lead pain experience and expression to conform to the social environment in which the child grows up.
6. Greater systematization in understanding of the processes whereby clinicians and others make judgments and treatment decisions concerning the care of children. Technological advances seem inevitable.
7. Greater consideration of the social ecological contexts of care provision, including more support for study of social determinants of pain and revision of health practitioner educational curricula to embrace psychosocial factors
8. Greater sensitivity and provision of access to care for vulnerable populations, including infants and children, and those substantial populations of people who are socially marginalized.

## 10 A FINAL ACKNOWLEDGEMENT

The foregoing is more self‐preoccupied than should be the case for scholarly expositions, perhaps justified by the task to which I was assigned, but at least there is room to recognize that full consideration of the issues raised here would entail exploration of a much more substantial corpus of scientific work generated by an increasing number of professionals and scholars. The recent volume “Social and Interpersonal Dynamics in Pain,” edited by Vervoort et al,^126^ provides state of the art reviews and effectively demonstrates how much progress has been achieved.
